# A novel sustained-release agent based on disulfide-induced recombinant collagen hydrogels for the prevention and treatment of *Schistosoma* infections

**DOI:** 10.1128/spectrum.03771-23

**Published:** 2024-12-19

**Authors:** Jie Wang, Lijun Song, Yuntian Xing, Yang Dai, Jinyuan Hu, Guoli Qu, Yongliang Xu, Xuren Yin, Derong Hang, Jianfeng Zhang, Chunrong Xiong, Liang Shi, Fei Xu

**Affiliations:** 1 National Health Commission Key Laboratory of Parasitic Disease Control and Prevention, Jiangsu Provincial Key Laboratory on Parasite and Vector Control Technology, Jiangsu Institute of Parasitic Diseases, Wuxi, China; 2 Ministry of Education Key Laboratory of Industrial Biotechnology, School of Biotechnology, Jiangnan University, Wuxi, China; University of Wisconsin-Madison, Madison, Wisconsin, USA

**Keywords:** recombinant collagen hydrogel, self-assembly, praziquantel, sustained drug release, schistosomiasis

## Abstract

Schistosomiasis is commonly managed using the praziquantel, but it is only effective against adult worms and duration of action is short. Liver fibrosis will worsen if eggs are still present after stopping treatment. Therefore, this study aimed to develop a sustained drug release system for effectively preventing and treating schistosomiasis. A disulfide bond-induced three-dimensional (3D) recombinant collagen hydrogel was developed for sustained praziquantel release. Three collagen sequences were developed based on the sequence for *Scl2* of *S. pyogene*, with different substitutions of residues for cysteine (S-VCL-S1, S-VCL-S2, and S-VCL-S3). Their properties were tested. Mice were infected with *Schistosoma japonicum* cercariae and treated either with praziquantel collagen hydrogel or niclosamide collagen hydrogel. The worm-killing effect was examined. The application of hydrogel-niclosamide on the skin for 24 h effectively prevented *Schistosome* cercariae from infecting mice and showed 70.95% and 81.73% reduction in the number of eggs and worms, respectively. The combined use of niclosamide and anti-cercariae cream showed 100% protection after 24 h. The hydrogel-praziquantel also showed a 100% reduction of worms and eggs after 24 h of subcutaneous injection. The subcutaneous injection of praziquantel after 28 days of infection showed 95.19% and 80.12% reduction of worm and egg counts, respectively, and the development of larvae was significantly slowed down. Liver analysis showed no infection after 7 days of treatment. These results suggest developing a novel type of sustained-release agent against schistosomiasis based on the recombinant collagen hydrogel that provides a potential new treatment for schistosomiasis.

**IMPORTANCE:**

This study introduces an new way for treating schistosomiasis: a special collagen hydrogel that gradually releases medication to treat schistosomiasis effectively. This innovation provides a promising way to treat schistosomiasis. It represents a significant step forward in the fight against this disease and offers hope for more effective and safer treatments in the future.

## INTRODUCTION

Schistosomiasis is a zoonotic disease caused by *Schistosoma* infection and seriously threatens human health and life (1, 2). Its symptoms include abdominal pain, enlarged liver, blood in stool and etc. *Schistosoma* cercariae penetrating the skin will cause reddening and itching (1, 2). Chronic schistosomiasis may lead to liver fibrosis and hepatic ascites. One of the most effective approaches to prevent the spread of schistosomiasis is eliminating the intermediate host (snails like *Oncomelania*). On the other hand, early treatment can prevent chronic infection. At present, niclosamide and praziquantel are widely used in controlling the transmission of *Schistosoma* and treating schistosomiasis and are among the drugs deemed essential by the World Health Organization (WHO) (3). Niclosamide kills *Oncomelania*, but it causes environmental pollution (4, 5). On the other hand, praziquantel, although less toxic, has a short *in vivo* half-life and only kills adult worms; therefore, it has limited effectiveness in the prevention and treatment of schistosomiasis (6). Therefore, the development of a novel sustained-drug release system is necessary to improve the management of schistosomiasis. In addition to controlled drug release, the developed system should increase the drug’s half-life, and the system’s components should be less toxic to the environment and safe for human use (6).

Hydrogels are soft polymeric materials comprised of hydrophilic natural or synthetic polymers and possess a 3D crosslinking network structure. Due to their high swelling properties, they can absorb large quantities of liquid between the polymer chains (7). Owing to their biocompatibility, biodegradability, high mechanical strength, stability, high drug-loading capacity, and sustained drug release, hydrogels are of great interest to the scientific community (8) and have been extensively used for drug delivery (9), wound dressing (10), tissue engineering (10), and other applications. A hydrogel facilitates the release of a drug encapsulated or entrapped in it according to variations in environmental conditions, like pH, temperature, and ionic strength or under the effect of external stimuli. According to the target applications, hydrogels are applied in different forms like injectables, patches, dressings, etc. Injectable hydrogels have attracted great attention for their simple preparation, accurate dosage, convenient administration, and little dependence on extrinsic factors. An injectable thermoresponsive polyethylene-poly(methide) amphiphilic block copolymers hydrogel was developed that showed extended and sustained drug release (11).

Hydrogels and drug-loaded hydrogels can also be used as antiparasitic owing to their high mechanical strength, excellent stability, large drug-loading capacity, and sustained and extended drug release (12, 13). The hydrogels currently in use are mostly based on natural proteins (e.g., animal gelatin) or ester alcohol copolymer, and, therefore, not suitable for sustained release of drugs *in vivo* because of the risk of contamination. Natural collagen-based hydrogels have been recently developed and used for antiparasitic drug delivery and benefit from high mechanical strength, excellent stability, large drug-loading capacity, and sustained and extended drug release. Gelatin, one of the materials of natural collagen hydrogel, forms a unique fibrous spatial network structure through self-assembly and is used for drug release, tissue regeneration, and wound healing (14). Compared with the natural collagen-based hydrogels, the recombinant collagen produced from the sequence of the collagen-like protein Scl2 of *Streptococcus pyogene* is expressed in large amounts in engineered bacteria and is correctly folded (15, 16). Furthermore, the recombinant collagen-based hydrogels have high purity, good biocompatibility, high biodegradability, and molecular designability and are free from pathogen contamination, thus paving the way for their use as biomedical materials (17). Nevertheless, the inability of the Scl2 protein to self-assemble and form collagen fiber or hydrogel limits its direct use as a biomaterial, and researchers are attempting to enable collagen to self-assemble into a hydrogel under specific conditions (18). In previous studies by the authors, the introduction of cysteine into the N and C terminal of *S. pyogene*’s collagen-like protein Scl2 promoted its self-assembly by forming disulfide bonds between cysteine residues and form a hydrogel, allowing the loading and release of drugs without any external crosslinking agent or polymer (19, 20).

Herein, cysteine residues were introduced at different sites, including the middle region, 1/3, and 2/3, regions of the polymer chain from the ends of the Scl2, and a self-assembled recombinant collagen hydrogel with controllable microstructure and mechanical properties was obtained by adjusting the number of cysteine residues and the ratio of the redox agents. The recombinant hydrogel was evaluated for *T*_m_ value, microstructure, mechanical strength, and drug release properties. The cytotoxicity of the recombinant collagen hydrogel and its effects on killing cercariae *in vitro* were also evaluated. Finally, this study investigated the prevention and treatment efficacy of the prepared hydrogel against parasitic disease in an animal model. The study showed that the recombinant collagen hydrogel demonstrated sustained drug release and effectively prevented schistosomiasis infection.

## RESULTS

### Design, expression, and purification of recombinant collagen hydrogel

The collagen coding sequence for *Scl2* of *S. pyogene* contains a CL sequence of globular domain V (VCL) and a trihelix domain Gly-Xaa-Yaa. A previous study showed that recombinant collagen hydrogel with cysteine introduced at both ends of VCL facilitates the formation of disulfide bonds *via* redox reaction, forming a hydrogel network (20). Based on the mechanical strength of the hydrogel obtained by Wang et al. (20), a series of alternative sequences was constructed, including S-VCL-S1, S-VCL-S2, and S-VCL-S3, based on S-VLC-S.

**Fig 1:**
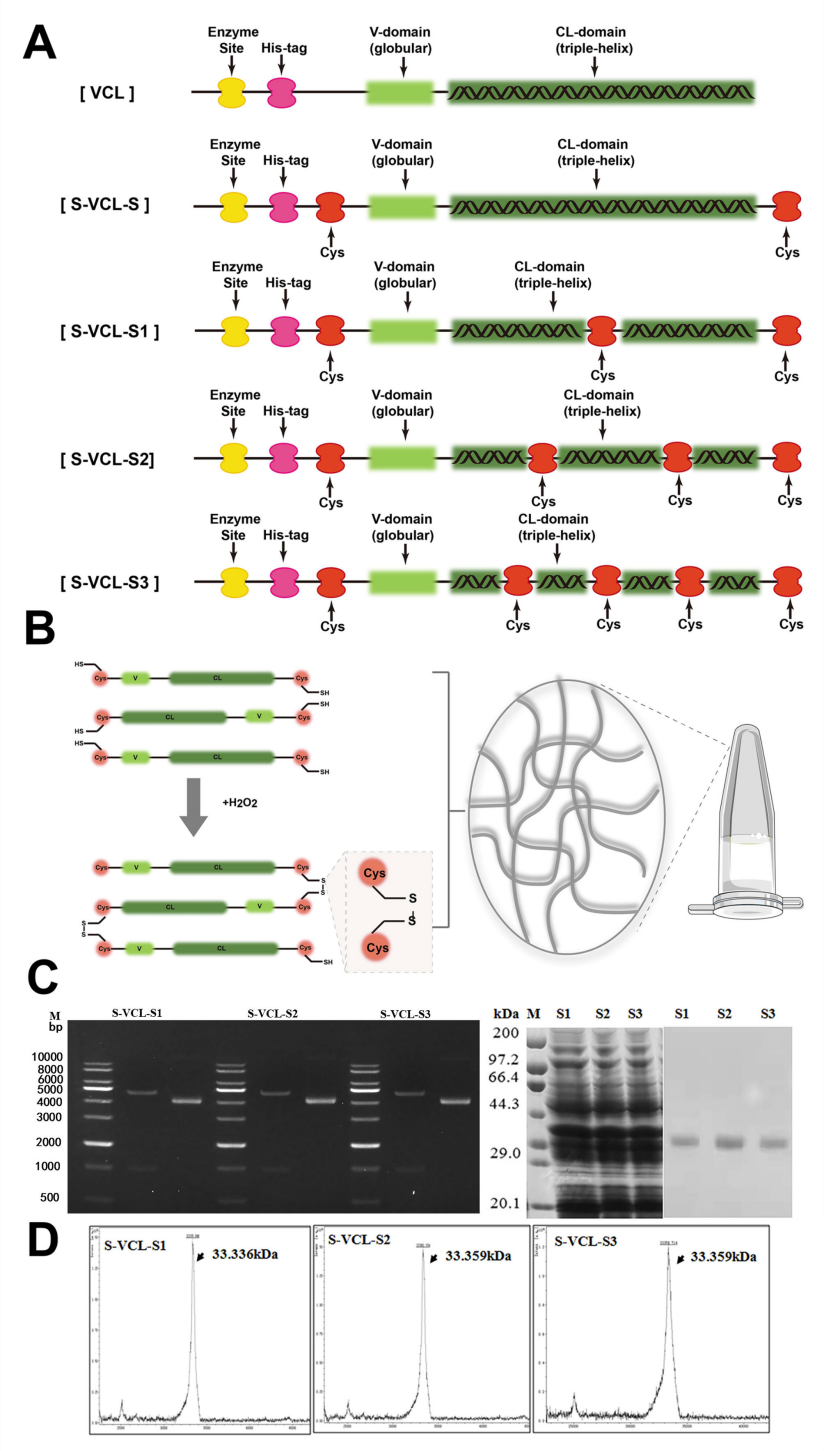
(**A**) Schematic illustration of the designed S-VCL-S1, S-VCL-S2, and S-VCL-S3 collagen protein with cysteine residues inserted at the N- and C- termini and the appropriate amino acids located in the middle and 1/3 and 2/3 of collagen are replaced by cysteine. (**B**) Molecular networks are created through the formation of the disulfide bonds between the cysteine residues. (**C**) The length of the nucleotide fragments after digesting the recombinant plasmids with BamHI and Nco I enzymes. M is the DNA marker. The left shows the intact pET-28a-S-VCL-S1-3 constructs, while the right shows the same constructs after double digestion with BamHI and Nco I. Molecular weights of proteins determined through SDS-PAGE analysis of the purified collagen proteins, S-VCL-S1-3. M shows standard protein marker; Left shows protein expression before purification of S-VCL-S1, SVCL-S2, and S-VCL-S3; Right shows S-VCL-S1, SVCL-S2, and S-VCL-S3 after purification. (**C**) MALDI-TOF mass spectrometry characterizing the molecular weights of S-VCL-S1, SVCL-S2, and S-VCL-S3 collagen.

In order to investigate whether the introduction of cysteine residues affects the triple helix structure and thermal stability of collagen, cysteine residues were introduced into the X and Y position of each G-X-Y in the Scl2 triple helix structure (Fig. 1A). Furthermore, to introduce cysteine into the N- and C-terminal of the VCL domain, the amino acid located in the middle and at 1/3 and 2/3 of CL was replaced with cysteine residues without reducing the *T*_m_ value of the protein. The threonine at position 121 was replaced by cysteine in S-VCL-S1. The alanine at position 76 and valine at position 154 were replaced by the cysteine in S-VCL-S2. The threonine at position 121, the alanine at position 76, and the valine at position 154 were replaced by the cysteine in S-VCL-S3. The design of the protein sequence is shown in Fig. 1A. All sequences contained a His6 tag in the N-terminal for protein purification (Fig. 1A). Herein, H_2_O_2_ induced the formation of intermolecular disulfide bonds between the inserted cysteines to form a hydrogel network (Fig. 1B).

The S-VCL-S1, S-VCL-S2, and S-VCL-S3 collagen were expressed in *Escherichia coli* BL21 (DE3) and purified using a HisTrap HP column. SDS-PAGE (Fig. 1C) and MALDI-TOF/TOF (Fig. 1D) analyses confirmed the molecular weight and purity of the protein, and the results showed that the molecular weight of S-VCL-S1-3 was about33.336 kDa. The error between the measured and theoretical values was within 0.05%.

### CD of collagen

**Fig 2:**
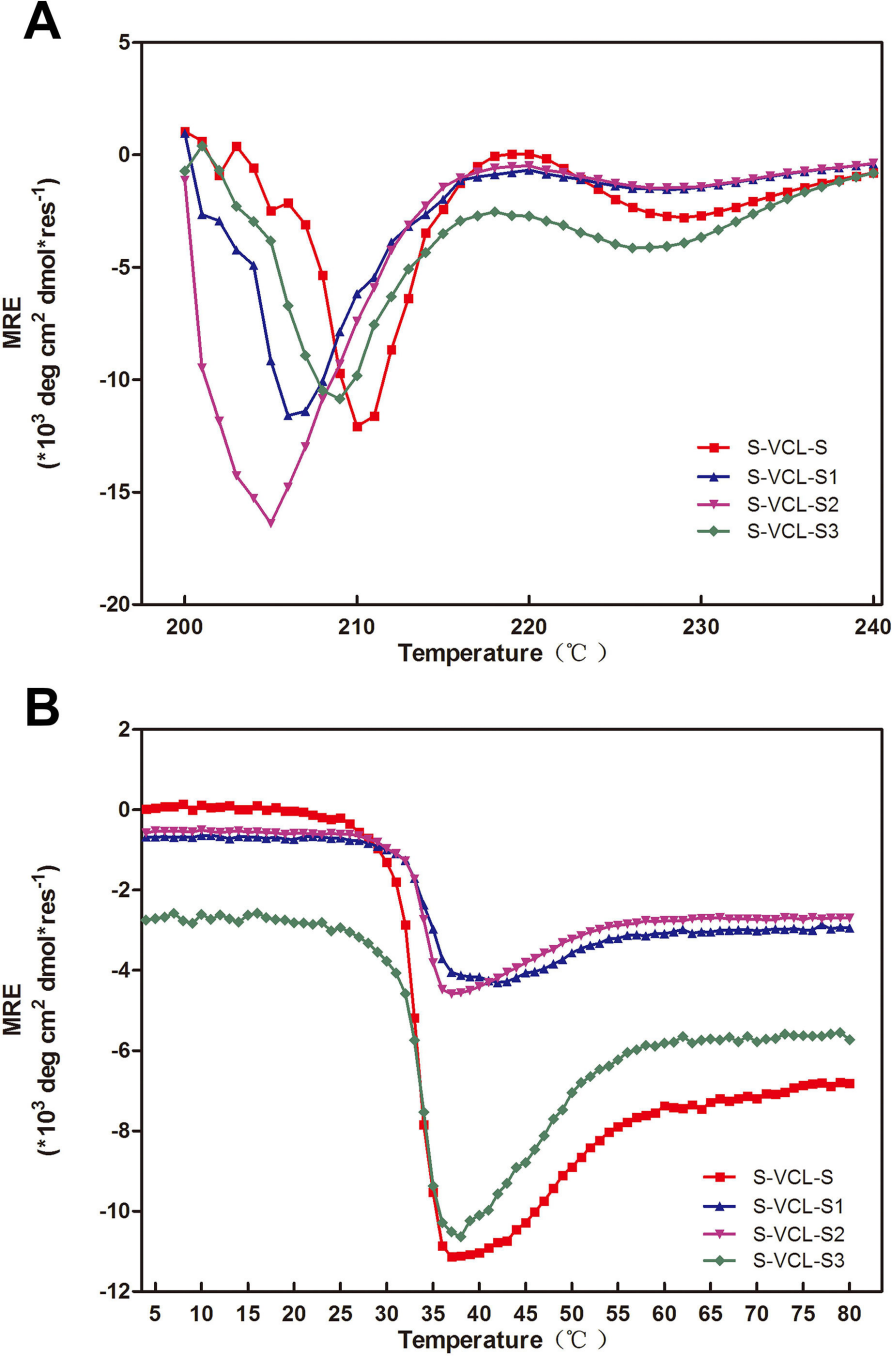
CD characterizations of the designed proteins. (**A**) Far-UV CD spectra of the collagen proteins and (**B**) thermal denaturation profiles of S-VCL-S, S-VCL-S1, SVCL-S2, and S-VCL-S3 at 1 mg/mL in 10 mM phosphate buffer (pH 7.4).

The secondary structure of the protein by circular dichroism (CD) spectrum was characterized at a low concentration (1 mg/mL) to evaluate the correctness of the triple helix structure of the S-VCL-S1-3 proteins, and the results are shown in Fig. 2. Compared with the S-VCL-S protein, the CD spectrum of S-VCL-S1-3 showed a typical triple helix spectrum at 200–240 nm with a positive peak at 220 nm and a minimum positive peak at 210 nm (Fig. 2A). The ellipticity difference was not significant at 220 nm among the three recombinant proteins. The average residual ellipticity (MRE) of S-VCL-S1 and S-VCL-S2 at 220 nm was nearly zero compared with the S-VCL-S3 protein (Fig. 2A). The results collectively demonstrate that each of the S-VCL-S1-3 proteins formed an α-helix and a triple helix similar to the S-VCL-S. The thermal stability of the S-VLC-S1-3 proteins was examined by monitoring the maximum strength of the triple helix (220 nm) at a heating rate of 0.1℃/min. The results showed that the melting transition of the S-VCL-S1-3 protein is S-shaped, which was similar to the melting curve of collagen and was consistent with the behavior of the S-VCL-S protein. The *T*_m_ value of S-VCL-S1-3 was 37℃ (Fig. 2B). Notably, the protein containing cysteine residues was as stable as the natural VCL, indicating that the introduction of cysteine residues into VCL may neither influence the formation of the secondary structure nor affect the stability of VCL.

### Rheological, mechanical, and morphological properties of the recombinant protein hydrogel

All the recombinant collagen proteins, i.e., S-VCL-S, S-VCL-S1, S-VCL-S2, and S-VCL-S3, formed transparent hydrogels and hung on the inverted centrifuge tube after being mixed with 0.1% H_2_O_2_ and incubation at 37°C for 15 min. The hydrogel remained intact for 3 days at 4°C (Fig. S1). The structural formula of the recombinant proteins was designed in a way that the number of cysteine residues was the highest in the S-VCL-S3 sequence (five cysteine residues), followed by S-VCL-S2 (four cysteine residues), S-VCL-S1 (three cysteine residues), and S-VCL-S (two cysteine residues).

**Fig 3:**
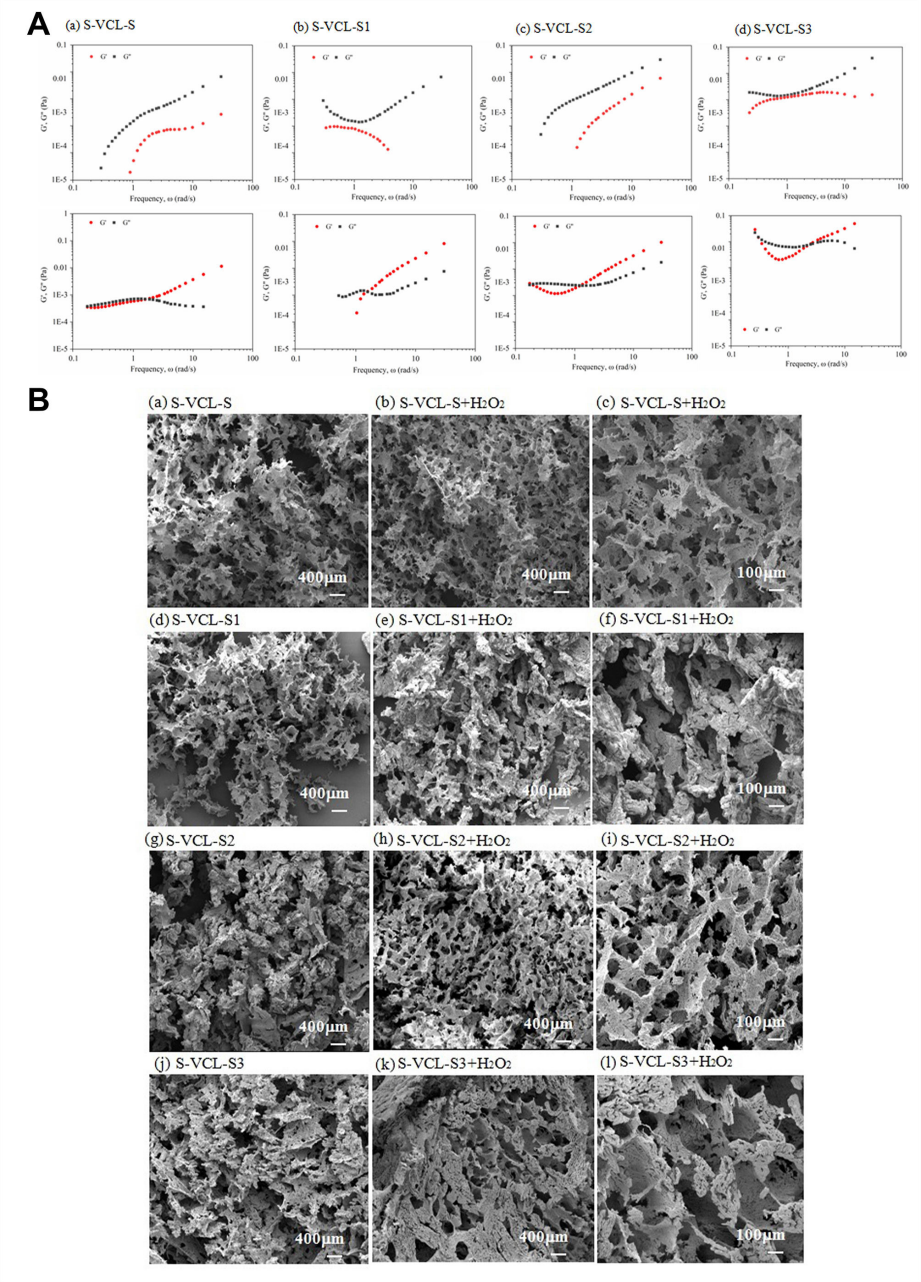
(**A**) Storage and loss moduli, G′ and G″, of S-VCL-S, S-VCL-S1, S-VCL-S2, and S-VCL-S3 hydrogels at 4 wt.% in 10 mM PBS (pH 7.4). (Above) 2 wt.%; (below) 4 wt.%. (**B**) SEM micrographs of the collagen-like proteins before and after the addition of H_2_O_2_. (A–C) S-VCL-S, (D–F) S-VCL-S1, (G–I) S-VCL-S2, and (**J–L**) S-VCL-S3. Scale bar = 400 µm for A, B, D, E, G, H, J, and K and 100 µm for C, F, I, and L. The concentration of S-VCL-S, S-VCL-S1, S-VCL-S2, and VCL-S3 is 4 wt.% with PBS or without 0.1 wt.% H_2_O_2_. PS: (**A–C**) S-VCL-S in Figure B as control here are from our previously published article titled “Recombinant collagen hydrogels induced by disulfide bonds.”

The formation of hydrogel was confirmed by measuring the energy storage modulus (G′) and consumption modulus (G″) of 4 wt.% of recombinant collagen with and without 0.1% H_2_O_2_ using a microheometer (Fig. 3A). The protein solution or hydrogel was mixed with fluorescent polystyrene beads and injected into the capillary space between the two slides. A high-speed camera was used to track and record the fluorescent beads’ movement to obtain the G′ and G″ of each recombinant protein hydrogel from the mean square displacement. The results showed that in the absence of H_2_O_2_, all 4 wt.% recombinant proteins were protein solutions with G ′< G″. On the other hand, all recombinant proteins formed hydrogels in the presence of H_2_O_2_. The S-VCL-S, S-VCL-S1, S-VCL-S2, and S-VCL-S3 proteins achieved the sol-gel transition points at scanning frequencies of 2.232, 1.682, 1.519, and 2.089 rad/s, respectively. All recombinant proteins created soft gels, and the modulus of S-VCL-S, S-VCL-S1, S-VCL-S2, and S-VCL-S3 proteins was found to be 0.0012, 0.0013, 0.0025, and 0.016 Pa, respectively. The mechanical strength of the hydrogels was, in order, S-VCL-S3 > S-VCL-S2 > S-VCL-S1 > S-VCL-S, indicating that the mechanical strength of the hydrogel was positively associated with the number of cysteine residues, which is potential because more cysteine residues provide a larger number of disulfide linkage sites on the surface of the proteins, thus increasing the binding stability and mechanical strength of hydrogel.

Compared with S-VCL-S in Fig. 3B a–c as control here are from our previously published article titled “Recombinant collagen hydrogels induced by disulfide bonds,” the introduction of different amounts of cysteine residues (2–5) into the recombinant collagen protein may influence the microstructure of the collagen micropore. The SEM micrographs of the internal porous microstructure of each recombinant collagen showed that the lyophilized S-VCL-S1-3 exhibited a loose porous structure in the absence of H_2_O_2_ (Fig. 3Ba, d, and h). After the addition of H_2_O_2_, the S-VCL-S1 protein formed a dense network structure (Fig. 3Be-f), whereas the S-VCL-S2 protein formed a honeycomb-like network structure with an aperture larger than S-VCL-S1 (Fig. 3Bh-i). On the other hand, the S-VCL-S3 protein presented a fibrous network structure with a larger micropore size than S-VCL-S2 (Fig. 3Bk-l). These results demonstrate that the microstructure of hydrogels containing H_2_O_2_ was distinct from the hydrogel without H_2_O_2_, and the number of cysteine residues significantly influences the hydrogel’s internal structure. These results further indicate that the number of cysteine residues in the collagen proteins determines the microporous structure of the self-assembled hydrogel.

All these results showed the successful formation of recombinant collagen hydrogel upon adding H_2_O_2_ into 4 wt.% protein solution, which improved the gelling properties of collagen. Furthermore, the introduction of cysteine residues improved the mechanical strength and porosity of the hydrogel. Compared with the S-VCL-S1 and S-VCL-S2 proteins, the S-VCL-S3 hydrogel was more rigid and possessed the largest pore size and was, thus selected for the subsequent experiments.

### Drug release behavior of the recombinant collagen hydrogel

**Fig 4:**
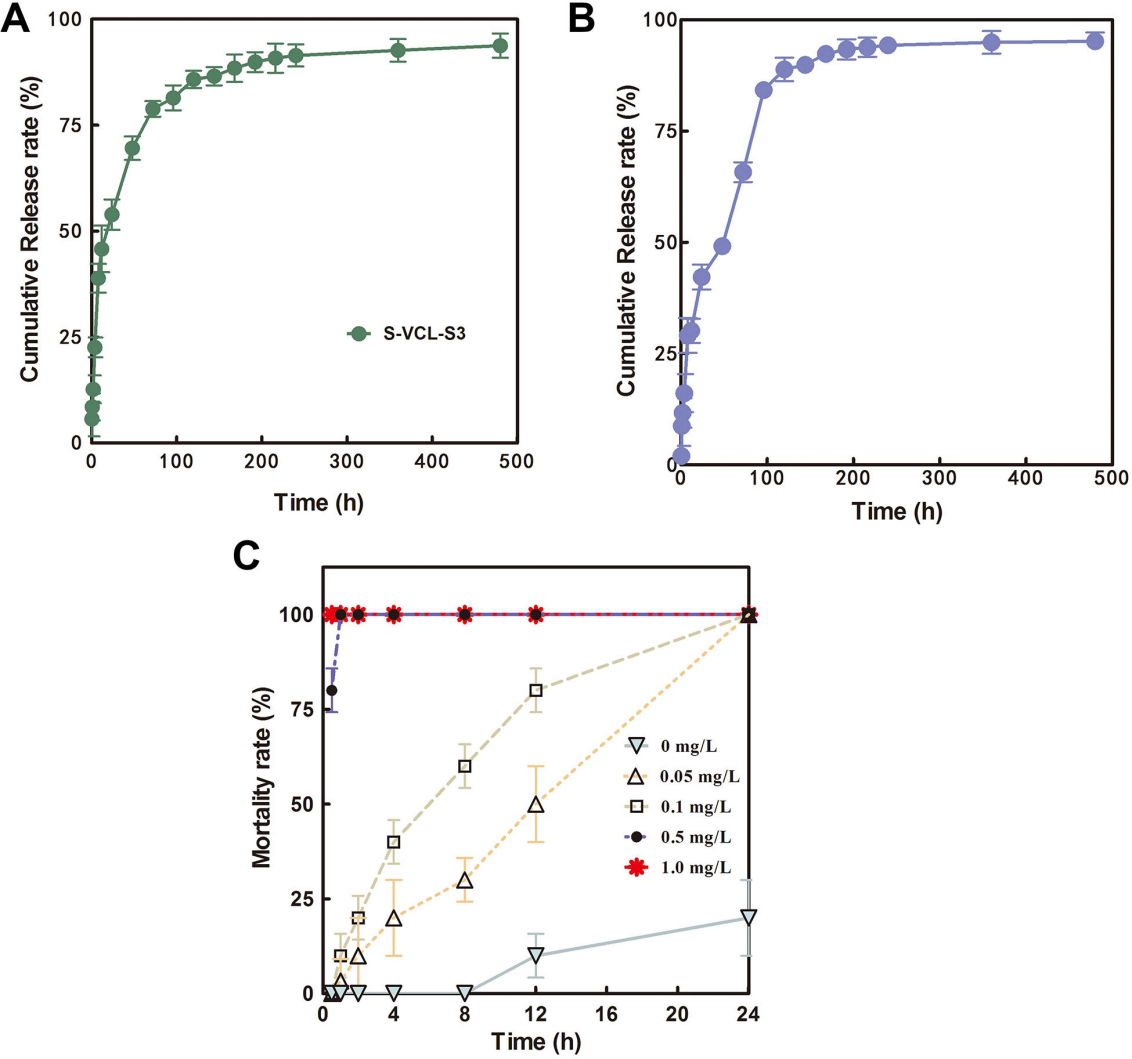
Sustained release of (**A**) niclosamide and (**B**) praziquantel from the recombinant collagen hydrogel S-VCL-S3. (**C**) Fitting curve of *S. cercariae* mortality at different time points and different concentrations of sustained-release niclosamide recombinant collagen hydrogel. The difference was considered significant at *P* < 0.05 (*n* = 3).

The drug release behavior of the S-VCL-S3 recombinant collagen hydrogels for cumulative release of niclosamide and praziquantel was determined (Fig. 4A and B, respectively). The S-VCL-S3 recombinant collagen hydrogel released 80% of niclosamide (Fig. 4A) and praziquantel (Fig. 4B) after 72 h, and the drug release lasted for 20 days, indicating a sustained release and suggesting its suitability as a drug delivery carrier.

### Effect of hydrogel-niclosamide sustained-release on cercariae

The killing effect of the sustained release of niclosamide from the S-VCL-S3 recombinant collagen hydrogel was determined on cercariae. A 0, 0.05, 0.1, 0.5, and 1 mg/L of niclosamide were loaded into the hydrogel, and the LC50 of the drug hydrogel on cercariae was calculated after 0.5, 1, 2, 4, 8, 12, and 24 h (Fig. 4C). The results showed that the S-VCL-S3 recombinant collagen hydrogel with niclosamide concentrations of 1, 0.5, 0.1, and 0.05 mg/L had a toxic effect on cercariae at all time points except after 0.5 h, and the mortality rate was positively correlated with the concentration of niclosamide (Table 1). The S-VCL-S3 recombinant collagen hydrogel containing 0.05 mg/L niclosamide killed all cercariae within 24 h, whereas the hydrogel containing 1 mg/L niclosamide killed all cercariae in 30 min. Cercariae penetrate human skin within 30 min after infection, indicating that this hydrogel could be applied as an alternative to the anti-cercaria cream.

**TABLE 1:** The mortality rate of *S. japonicum* cercariae in niclosamide sustained-release agents with recombinant collagen hydrogel S-VCL-S3 %

Niclosamide concentration in Recombinant collagen hydrogel (mg/L)	Mortality rate (%)						
	30 min	1 h	2 h	4 h	8 h	12 h	24 h
0	0	0	0	0	0	10	20
0.05	0	3.33	10	20	30	50	100
0.1	0	10	20	40	60	80	100
0.5	80	100	100	100	100	100	100
1	100	100	100	100	100	100	100

### Effect of hydrogel-niclosamide sustained-release on cercariae infection in animals

**Fig 5:**
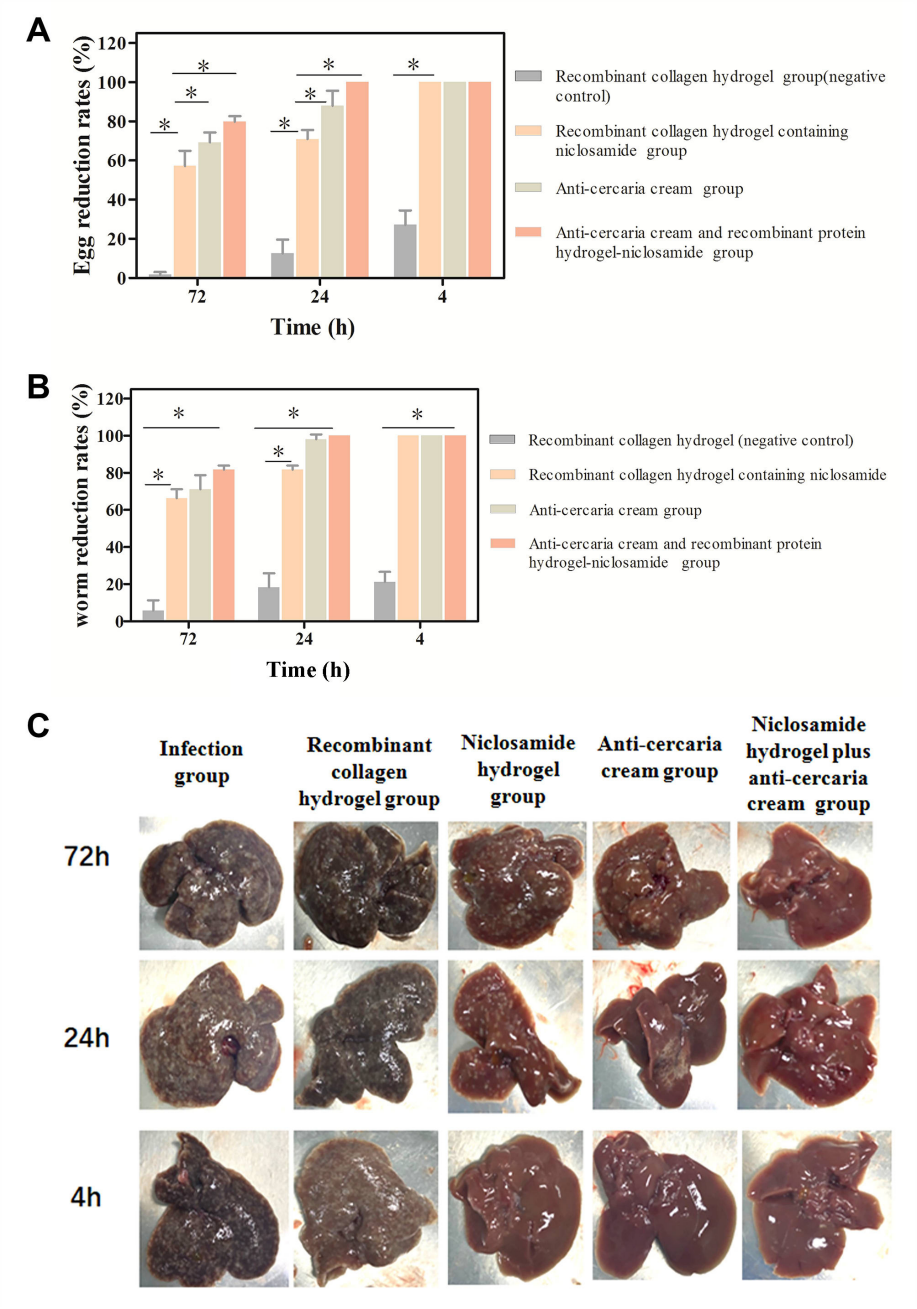
(**A**) Egg and (**B**) worm reduction rates (%) of recombinant collagen hydrogel with sustained niclosamide release applied to the abdomen of mice before 4 to 72 h of *S. cercariae* infection. (**C**) Digital images of liver egg of mice coated with recombinant collagen hydrogel niclosamide sustained-release agent on abdomen before 4 to 72 h *S*. *cercariae* infection liver analysis.

The subsequent animal experiments tested the S-VCL-S3 recombinant collagen hydrogels with sustained-drug release behavior. The anti-cercariae efficacy of the hydrogel-niclosamide sustained release was determined by counting the number of eggs and adult worms in the liver of mice (Fig. 5A and B), respectively. The worm and egg reduction rates were increased after 72, 24, and 4 h in the recombinant collagen hydrogel group (Tables S1 and S2). Similar results were shown in the niclosamide hydrogel group and the group treated with anti-cercaria cream. The mice smeared with niclosamide hydrogel + anti-cercaria cream after 4 h before infection with cercariae showed no egg nodules in the liver. When smearing the drugs after 24 h before infection with cercariae, the egg nodules were significantly reduced compared with the infection group; however, there were still 10%–20% eggs. When smearing the drugs after 72 h before infection with cercariae, the insecticidal effect of the drugs was dramatically decreased, and the worm and egg reduction rates decreased to 60%–70%. If the hydrogel-niclosamide was smeared on mice and subsequently smeared the same mice with anti-cercariae cream, the average worm reduction rates were increased after 72, 24, and 4 h. These two groups’ worm and egg reduction rates were significantly different from the negative control (*P* < 0.05). These results show that the hydrogel-niclosamide sustained release agent has a significant effect in fighting the cercariae infection. After 24 h, the hydrogel agent was smeared on the mice. The worm reduction rate was increased from 80% to 100% in the infection group when the hydrogel was combined with the anti-cercariae cream. There were no egg nodules in the livers of this group (Fig. 5C). These data suggest that the hydrogel-niclosamide sustained release agent could effectively treat the anti-cercariae cream.

### Prevention and therapeutic effects of hydrogel-praziquantel sustained release agent *in vivo*

**Fig 6:**
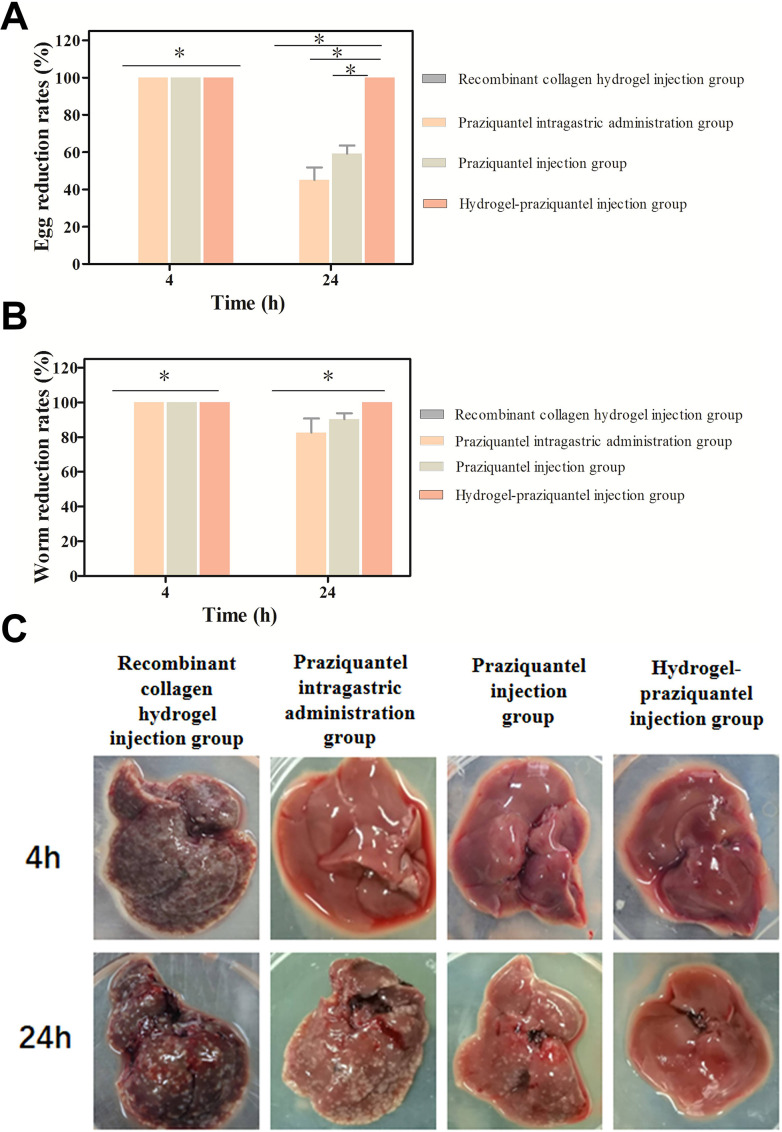
(**A**) Egg and (**B**) worm reduction rates (%) in mice treated with subcutaneous injection of recombinant collagen hydrogel containing praziquantel sustained-release agent 4 to 24 h before *S. cercariae* infection. (**C**) Digital images of liver egg of mice coated with recombinant collagen hydrogel niclosamide sustained-release agent on abdomen before 4 to 72 h *S*. *cercariae* infection liver analysis.

**Fig 7:**
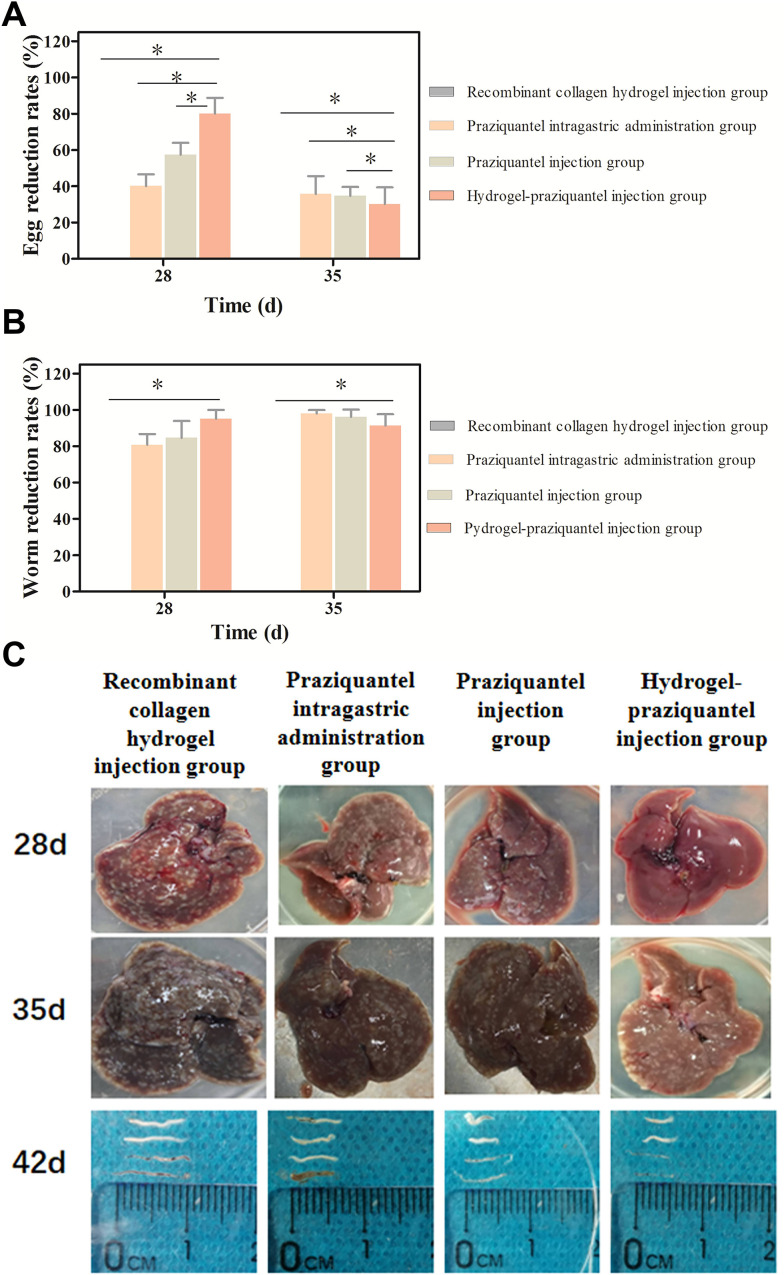
(**A**) Egg and (**B**) worm reduction rates (%) in mice treated with subcutaneous injection of recombinant collagen hydrogel containing praziquantel sustained-release agent after 28 and 35 days of *S. cercariae* infection. (**C**) Digital images of the liver eggs of mice coated with recombinant collagen hydrogel praziquantel sustained-release agent after 28 and 35 days of *S. cercariae* infection.

The prevention and therapeutic effects of the hydrogel-praziquantel sustained release agent on the infection of *S. japinicum* cercariae were determined *in vivo*, and the results are shown in Fig. 6 and 7, respectively. The results showed that the worm and egg reduction rates in the mice injected with drug-free recombinant collagen hydrogel were both 0% but reached 100% after subcutaneous injection of praziquantel without collagen hydrogel, hydrogel-praziquantel, and intragastric administration of praziquantel (Fig. 6A through C). Importantly, the egg reduction rate in the hydrogel-praziquantel subcutaneous injection group was significantly different from the recombinant collagen group, the praziquantel administration group, and the praziquantel subcutaneous injection group in mice treated 24 h before cercariae infection (*P* < 0.05) (Table S3). The results show that the drug gavage and injection groups had a 100% anti-infection effect when the mice were treated 4 h before infection. However, due to the short half-life of praziquantel, the killing effect of the drug on cercariae was significantly decreased when the treatment was given 24 h before infection. Thus, the worm and egg reduction rates of the praziquantel intragastric and injection groups decreased significantly. On the other hand, the hydrogel-praziquantel enabled sustained release, and the worm and egg reduction rate reached 100%, indicating its effectiveness in preventing the *S. japonicum* cercariae infection.

The results of the therapeutic effect of the subcutaneously injected hydrogel-praziquantel sustained release agent in mice 28 and 35 days after cercariae infection are shown in Fig. 7; Table S4. The results showed that the worm and egg reduction rates in mice injected with drug-free recombinant collagen hydrogel were both 0%, reaching 95.19% and 91.35% after 28 and 35 days, respectively, in the group treated with subcutaneous injection of hydrogel-praziquantel (Fig. 7C). After 28 and 35 days of cercariae infection, the worm and egg reduction rates in all experimental groups significantly differed from those of the control group (*P* < 0.05). It is worth noting that the intragastric administration of praziquantel had a great insecticidal effect, and the worm reduction rate was relatively high both after 28 and 35 days of cercariae infection, while the egg reduction rate was relatively low, indicating that praziquantel is not suitable for early intervention and treatment of the infected animal. These results could be explained by the short half-life of praziquantel, which is active when worms are not completely mature, thus potentially leading to the worms’ lack of response to the drug. After 28 days of cercariae infection, the subcutaneous administration of praziquantel slightly increased the worm and egg reduction rates; however, its effects remained unsatisfactory for the prevention and treatment effect. These data suggest that the hydrogel-praziquantel sustained-release agent has an excellent protective effect on the liver of mice and is effective in preventing and treating schistosomiasis infection.

## DISCUSSION

Schistosomiasis seriously threatens human health and, thus, requires effective prevention measures. The cercariae are encapsulated and engulfed by many leukocytes as part of the inflammatory response process when they invade the body (21). Studies have shown that the penetration of cercariae in the skin is prevented by niclosamide and anti-cercaria cream due to their pharmacological and anti-inflammatory effects (22–24). However, niclosamide and praziquantel have several limitations (6, 25). It necessitates the development of sustained-release biomaterials with natural origin and safe sustained-release characteristics that can be coated and injected into the body to overcome the limitations mentioned above. This study aimed to find a safe and effective biological sustained-release agent for the prevention and early treatment of schistosomiasis infection.

Herein, the three recombinant collagen proteins, including S-VCL-S1, S-VCL-S2, and S-VCL-S3, developed by introducing cysteine residues into the middle, 1/3, and 2/3 of the collagen gene sequence of *S. pyogene*, retained the characteristics of natural collagen. The stimulated disulfide bonds formed a hydrogel network under redox conditions and showed no cytotoxicity in accordance with previous studies (20, 26). Among them, S-VCL-S3 recombinant collagen hydrogel showed the best gelling performance and formed stable natural collagen hydrogel through self-assembly and demonstrated excellent drug release performance for niclosamide and praziquantel. The S-VCL-S3 gel, therefore, could be an ideal drug release agent with promising application potential.

The killing effect of the S-VCL-S3 hydrogel-niclosamide on cercariae showed that the hydrogel effectively killed cercariae within 24 h. The smearing of 1 mg/L niclosamide S-VCL-S3 hydrogel to the abdomen of mice significantly decreased the infection rate. Both the worm and egg reduction rates reached 100% after 24 h when the treatment was combined with the anti-cercaria cream application. These results demonstrate that S-VCL-S3 recombinant protein hydrogel containing niclosamide effectively inhibited the infection of cercariae and is effective in killing cercariae, which is in accordance with a previous study demonstrating multiple mechanisms for preventing infections (27).

The S-VCL-S3 hydrogel-praziquantel sustained release agent (S3 hydrogel-praziquantel) showed preventive and therapeutic effects on schistosomiasis in infected mice. The subcutaneous injection of S-VCL-S3 hydrogel containing praziquantel completely removed any eggs or worms. Due to the short half-life of praziquantel, the killing efficacy of adult worms was limited, and praziquantel could not kill all new adult worms. It was hypothesized that the adult epidermis is not fully mature at this time, voiding the drugs’ killing effect or making the *Schistosoma* not sensitive to the drug, which explains why the oral administration or injection of praziquantel without a slow-release effect cannot prevent the infection. In addition, the insecticidal treatment is inefficient before the adult worm oviposition. The S3 hydrogel-praziquantel effectively solved the problem of liver metabolic burden due to the repeated administration of the drug. Its sustained-release effect and continuous drug release effectively killed adult worms by early intervention after 24 h of infection and 28 days after infection. The results show that the worm and egg reduction rates in the injection group dramatically decreased and that this treatment option protected the host liver and effectively prevented and treated the schistosomiasis infection.

In conclusion, the recombinant collagen drug hydrogel has an obvious effect in reducing the *S. japonicum* cercariae infection, the adult worm rate *in vivo*, and the egg rate in the liver, demonstrating that the recombinant collagen developed in this study is suitable for drug release and presents broad potential uses in preventing cercaria infection. It was noted that recombinant collagen was more soluble in an environment with water and temperature higher than 37°C. A study reported the development of natural 3D biomaterials with better temperature tolerance and rigidity, which can be combined with drugs to prevent and control schistosomiasis (9). Herein, it was concluded that the collagen hydrogel could serve as a sustained-release material to prevent schistosomiasis infection, paving the way to using natural collagen hydrogel and providing novel insights into treating schistosomiasis.

In this study, recombinant collagens that formed hydrogels by disulfide bonds crosslinking self-assembly were constructed *via* gene modification by introducing cysteine into the multiple specific sites in VCL. The recombinant collagen hydrogel showed good mechanical strength and biocompatibility, which solves the self-assembly problem and presents a great drug release ability, with a release time of 20 days and a release rate of 80%. To the best of the authors’ knowledge, this is the first report of using recombinant collagen hydrogel as a drug release agent to prevent and treat parasitic infections.

Of course, this study has limitations. It was performed in animals, and translation in humans must be tested in clinical trials. Toxicity was not examined *in vitro*. Finally, although several species of *Schistosoma* can infect humans, only *S. japonicum* was tested in the present study.

We demonstrated the good therapeutic effect of the developed hydrogels against cercariae and parasitic infections. The findings of this study pave the way for the development of injectable recombinant collagen hydrogel sustained-release agents *in vivo*.

## MATERIALS AND METHODS

### Reagents and equipment

Niclosamide (CAS:50-65-7; purity ≥98%) (Sigma, USA) solution was prepared with high-performance liquid chromatography (HPLC)-grade dimethyl sulfoxide (DMSO). The anti-cercariae cream was obtained from the Jiangsu Institute of Parasitic Diseases. Other reagents were purchased from the following manufacturers: metaldehyde 60 GR (Guangdong Nongmi Biotechnology Co., Ltd., China), cyano-4-hydroxycinnamic acid (CHCA) (Billerica, USA), acetonitrile and trifluoroacetic acid (TFA), methanol (HPLC grade) (Sigma, USA), and pure water (Millipore, USA). The pieces of equipment were purchased from the following manufacturers: electronic balance and pH meter (Mettler-Toledo, USA), constant-temperature water bath (Dongxing Building Materials Testing Equipment Co., Ltd., China), ARES-G2 rheometer (TA Instruments, New Castle, DE), Hitachi SU1510 Scanning Electron Microscope (Tokyo, Japan), FreeZone Plus 6L freeze-dryer (Labconco, USA), microplate reader (Thermo, USA), fluorescence confocal microscope (Leica, USA), and Agilent 1260 high-performance liquid chromatograph (Agilent, USA).

### Molecular design, construction, expression, purification, and characterization of the recombinant collagen

#### Recombinant collagen: molecular design of the collagen polymers

The collagen sequences containing the GFPGER region were designed using the Gene Designer software, as reported previously (20). Cysteine residues were introduced at the two end sites of the collagen sequence to enable oxidative crosslinking. The protein polymers are shown in Fig. 1A and B.

#### Recombinant collagen: plasmid construction

The synthesized S-VCL-S1, S-VCL-S2, and S-VCL-S3 sequences were digested by *BamH* I and *N*co I (New England Biolabs, USA) and then cloned into the pET-28a vector (GENEWIZ, China) for protein expression according to a previously reported protocol (20). *E. coli* DH5α cells and LB culture medium (containing 1% tryptone, 0.5% yeast extract, and 1% sodium chloride) were used for plasmid construction and amplification.

#### Recombinant collagen: recombinant protein expression

For protein expression, the S-VCL-S1, S-VCL-S2, and S-VCL-S3 sequences were transformed into *E. coli* BL21 (DE3) cells, as reported previously (20). Briefly, a single bacterial colony was cultured overnight in 4 mL LB medium with 50 µg/mL kanamycin (37°C and 220 rpm shaking). The overnight culture was diluted with fresh LB medium containing kanamycin at a 1:100 ratio and incubated in a shaker at 220 rpm at 37°C until the OD_600_ reached 0.6–0.8. After that, isopropyl β-d-1-thiogalactopyranoside (IPTG, 1 mM) was added and incubated for 4 h to induce protein expression.

#### Recombinant collagen: recombinant protein purification

The recombinant proteins were purified according to the protocol described in a previous study by the authors (20). After centrifugation at 8,000 rpm, the bacterial pellet was resuspended in binding buffer (20 mM NaPO_4_, 500 mM NaCl, 10 mM imidazole, pH 7.4) at 4°C and ultra-sonicated for 30 min in ice water. They were subsequently centrifuged at 12,000 rpm for 20 min at 4°C to remove bacterial debris, and the supernatant was filtered with a 0.45 µm microporous membrane. The proteins were purified using a 5 mL HisTrap HP column (GE Healthcare) pre-equilibrated with the binding buffer, eluted with increasing concentrations of imidazole (100, 200, 250, and 400 mM, 6 min for each treatment), and then desalted to remove imidazole using a 5 mL Hitrap Desalting Column (GE Healthcare) against the pure deionized water or 10 mM phosphate buffer saline (PBS). The protein expression was determined by SDS-PAGE analysis.

#### Recombinant collagen: CD spectroscopy

An Applied Photophysics Chirascan with a Peltier temperature controller (UK) was used for Far-UV CD spectra capture. Before the examination, a 1 mg/mL collagen solution (in 10 mM phosphate buffer, pH 7.4) was equilibrated for 24 h at 4°C. The scanning range was between 190 and 260 nm, with 1 nm steps and 5 s average time. The melting experiments were monitored at 4–80°C at 220 nm, with a 1°C rise in temperature every 6 min and >8 s samples’ equilibration at each temperature. *T*_m_ was calculated from the first derivative of the melting curves.

#### Recombinant collagen: observation of cysteine-based disulfide bond crosslinks

Compared with the control that only contained 90 µL of 4 wt.% S-VCL-S1, S-VCL-S2, and S-VCL-S3 solution, a 10 µL of 0.5%–1% H_2_O_2_ was added to the test tube, mixed gently, and incubated for 30 min at 37°C to allow the formation of disulfide bond crosslinks. After gel formation, the glass tube was turned upside down to observe the crosslinking status. If the polymer molecules formed a hydrogel by crosslink, they showed limited mobility and remained at the bottom of the tube. On the other hand, the gel formation could be monitored when it flowed down to the tube wall by a photographic camera.

#### Recombinant collagen: mass spectroscopy

The matrix-assisted laser desorption ionization (MALDI) spectrum was acquired by a MALDI- time-off flight (TOF)/TOF (Bruker Daltonics Corporation, Billerica, MA, USA) equipped with a 355 nm Smart beam II. The MALDI-TOF laser had a 450-ns delay pulsed ion extraction with an accelerating voltage of 19.5 kV. The attenuator offset of the laser was set at 30% according to the software instructions. Purified water was obtained using a Milli-Q apparatus (Millipore, USA). A 2 µg/µL protein solution was prepared by dissolving the protein in purified water, and then 1 µL of protein solution was applied on a 384-spot stainless steel MALDI plate. The solution was mixed with 1 µL of 5 µg/µL CHCA dissolved in 30% acetonitrile/H_2_O (vol/vol) and 0.1% TFA. The dried samples were analyzed using MALDI-TOF/TOF MS.

#### Recombinant collagen: microrheology

The stress relaxation modulus G(ϖ) was used to describe soft materials with complicated structures and characteristic lengths and time scales, such as gel solutions. Fluorescence polystyrene beads (Φ1.0 μm, Invitrogen, USA) were added to the protein solution at 4 wt.%. Beads in the field with >30 particle density were examined to observe the fluorescence by inverted microscopy (Leica, Germany). The trajectories of the 1 µm fluorescent polystyrene beads were tracked and analyzed using a CCD camera (QCAM QImaging, CAN), and the mean square displacement (MSD) was defined to be a function of time (28, 29). The location of a specific bead was determined by ~250 image trajectories recorded by the IDL image analysis software. MSD was defined as a lag time function, and G′ and G″ were set based on the Generalized Stokes-Einstein correlation (30).

#### Recombinant collagen: scanning electron microscopy

The 4 wt.% protein solution of S-VCL-S1, S-VCL-S2, and S-VCL-S3 was mixed with 0.1 wt.% H_2_O_2_ to prepare the hydrogels, which were then lyophilized *via* a FreeZone Plus 6L freeze-dry system (Labconco, USA). The hydrogels were subsequently sputter-coated with a thin palladium/gold alloy layer. Microstructure imaging was performed using a Hitachi SU1510 SEM (Tokyo, Japan).

### Preparation of drug collagen hydrogel and determination of sustained release of niclosamide

Niclosamide (5 µL, 1 mg/mL) was mixed with 90 µL of 4 wt.% protein solution, followed by adding 5 µL of 1% H_2_O_2_ into the 96-well plate. The plate was incubated at 4°C for 7 days to form the hydrogel. The surface of each well was covered with 200 µL of PBS to collect the released niclosamide. The content of niclosamide in PBS solution was detected at 0, 1, 2, 4, 6, 8, 12, 24, 36, 48, 72, 96, 144, 168, 192, 216, and 240 h. At each time point, 10 µL of solution was collected, and the well was replenished with equal volumes of double-distilled water (ddH_2_O). The solution collected at each time point was diluted to 1:50 with ddH_2_O. The samples were analyzed by HPLC with a ZORBAX SB-C18 column (4.6 × 250 mm, particle size 5 µm). The mobile phase was 90% methanol in water, pH 4. The column was kept at 25°C, the flow rate was 1 mL/min, the detection wavelength was 330 nm, and the injection volume was 5 µL. A 60 mg/L niclosamide/methanol solution was prepared and diluted to 30, 6, 3, 0.6, and 0.3 mg/L standard solutions, and the standard curve of niclosamide concentration was determined according to the chromatographic conditions. A linear regression analysis of the chromatographic peak area with the concentration was performed. The regression equation was *y* = 19.668*x*−3.7227, *R*^2^ = 0.9998, showing a good linear relationship. Standard solutions of 60, 6, and 0.6 mg/L were prepared, the measurements were repeated seven times, and precision was calculated. The RSD of 60, 6, and 0.6 mg/L were 0.68%, 0.62%, and 2.13%, respectively.

### Preparation of drug collagen hydrogel and determination of sustained release of praziquantel

Praziquantel (10 µL, 3 mg/mL) was mixed with 90 µL of 4 wt.% protein solution containing H_2_O_2_ in a 96-well plate. The plate was incubated at 4°C for 7 days to form the hydrogel. The surface of each well was covered with 200 µL PBS to collect the released praziquantel. The content of praziquantel in PBS solution was detected at 0, 1, 2, 4, 6, 8, 12, 24, 36, 48, 72, 96, 144, 168, 192, 216, and 240 h. Then, 10 µL of solution was collected at each time point, and the wells were replenished with equal volumes of ddH_2_O. The solution collected at each point was diluted to 1:50 with ddH_2_O. The samples were centrifuged at 8,000 rpm for 20 min, and the supernatant was transferred into a new tube. The samples were analyzed by ultra-performance liquid chromatography (UPLC) (SCIEX TRipley Quad TM 5500^+^) with a 1.8 µm T3 (50 mm × 21 mm). The mobile phase was 0.1% formic acid in acetonitrile with the following gradient: initial ratio of water to acetonitrile 90:10, 0.3 min 90:10, 1.5 min 5:95, 3.9 min 5:95, 4.0 min 90:10, 5 min 90:10, and the flow rate was 0.4 mL/min. The mass spectrum conditions were curtain gas (CUR): 40 psi, ion spray voltage (IS): 4,500 V, temperature (TEM): 450°C, collision gas (CAD): 7 psi, ion source gas 1 (N2), 50 Arb, and ion source gas 2 (N2), 50 Arb. Praziquantel (10.01 mg) was dissolved in 10 mL methanol to prepare the 1.001 mg/mL mother solution for praziquantel. Different concentrations of standard praziquantel solution, including 0.5, 1, 2, 5, 10, 20, 50, 100, 200, and 500 ng/mL, were prepared through step-by-step dilution. The standard curve of praziquantel concentration was determined according to the chromatographic data, and a linear regression of the chromatographic peak area with the concentration was performed. The regression equation was *y* = 19.668*x*−3.7227, *R*^2^ = 0.9998, showing a good linear relationship. For sample preparation, after the sample was uniformly swirled with a vortex, 10 µL of the sample was diluted into 1 mL with ddH_2_O_2_ to obtain a 1:100 dilution. Then, 20 µL of solution was diluted into 1 mL with ddH_2_O_2_ to obtain a 5,000-fold dilution. If the concentration exceeded the linear range, it was diluted until it was in the appropriate range by using the stepwise dilution method. Then, UPLC detection was conducted.

### *In vitro* toxicity experiment for cercariae

A 0.1 µL volume of niclosamide solution (1 mg/mL) was mixed with the recombinant collagen hydrogel (95 µL 4 wt.% hydrogel, 5 µL of 1% H_2_O_2_) in a 96-well plate. The plate was incubated at 4°C in the dark for 7 days to form the hydrogel. The surface of each well was covered with 200 µL PBS to collect the released niclosamide. The oncomelania containing cercariae was put into the dechlorinated water to release cercariae 6 h before the experiments under fluorescent light at 25°C. A total of 10 cercariae were put into each well, and the mortality of cercariae in the well was detected at 0.5, 1, 2, 4, 6, 8, 12, and 24 h.

### Animal experiments

#### Schistosoma and animals

**Fig 8:**
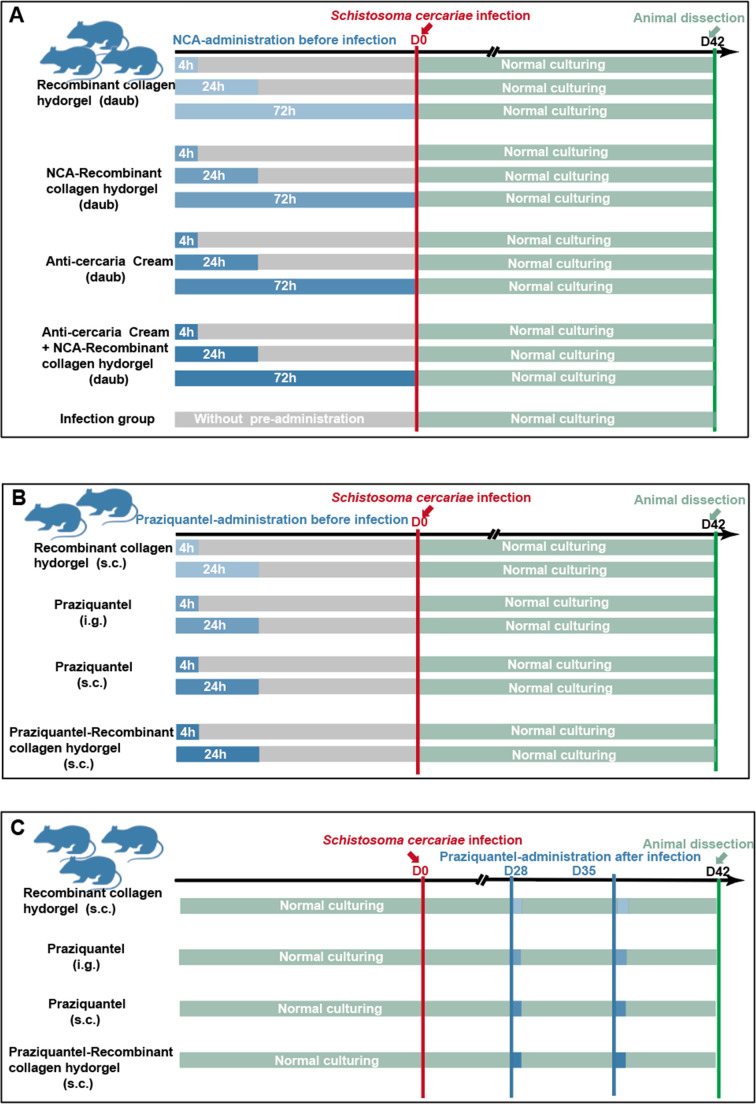
Study workflow. (**A**) The schematic diagram for different agents, including niclosamide sustained-release agents, was applied to the abdomen *in vitro* before 4–72 h of infection. (**B**) The schematic diagram for different agents, including S-VCL-S3 recombinant collagen hydrogel and praziquantel sustained-release agents, was administered subcutaneously before or at 4 or 24 h of infection. (**C**) The schematic diagram for different agents, including S-VCL-S3 recombinant collagen hydrogel and praziquantel sustained-release agents, was administered subcutaneously at 28 or 35 days of infection.

Fig. 8 presents the study workflow. The cercariae of *Schistosoma japonicum* were obtained from the Institute of Parasitic Diseases (Jiangsu Province). The purchased ICR mice (Yangzhou University, China) were 5–6 weeks old male and weighed 35.7 ± 0.69 g. The mice were allowed to eat a normal pellet diet and drink water freely for 3 consecutive days and then were stopped. The feces and food residues were collected 24 h before the experiment. During the experiment, only pure water was supplied (at 27°C).

All animal experiments were performed according to the guidelines of the Research on Schistosomiasis control in Jiangsu Province (approval #JIPD-2021-012). The ethics committee determined the number of animals used. According to the *E*-value method, the calculated *E*-value for each animal experiment was 20, which falls within the 10–20 range, indicating an adequate sample size for animal studies (31).

#### Prevention group by smearing

The mice were randomly divided into four groups of six male mice each and treated with 100 μL of each substance applied to their depilated abdomens (1.5 × 1.5 cm): (a) S-VCL-S3 recombinant collagen hydrogel, (b) recombinant collagen hydrogel containing niclosamide, (c) anti-cercaria cream, (d) a combination of anti-cercaria cream and S-VCL-S3 recombinant protein hydrogel-niclosamide, or (e) water (control group). At 72, 24, or 4 h after application, the mice were infected with 35 *Schistosoma japonicum* cercariae by pressing the slide containing cercariae against their depilated abdomens for 20 min. Mice in the infection group were infected directly with 35 *S. japonicum* cercariae. The water group served as the control.

#### Prevention group by injection or gavage

Ten milliliters of 2.5% polyoxyethylene castor oil was added to 15 tablets of praziquantel (0.2 g each). The mixture was ground into a ball mill at 190 rpm per minute for 1 h. The final volume was adjusted to 20 mL by adding polyoxyethylene castor oil to prepare the mother solution of praziquantel with a final concentration of 150 mg/mL. Mice with depilated abdomens were randomly divided into four groups of six mice each: (i) a control group receiving an injection of S-VCL-S3 recombinant collagen hydrogel; (ii); a praziquantel injection group; (iii) a praziquantel intragastric administration group; and (iv) a group receiving S-VCL-S3 recombinant collagen hydrogel containing praziquantel. Praziquantel was administered at 300 mg/kg body weight by gavage or subcutaneous injection in groups b and c, respectively. In group d, praziquantel wrapped with recombinant collagen hydrogel was administered at 300 mg/kg body weight by injection, while the control group received the same volume of recombinant collagen hydrogel only. After 4 and 24 h following gavage or subcutaneous injection, the mice were infected with 35 *S. japonicum* cercariae by pressing a slide containing cercariae against the depilated abdomen of each mouse for 20 min.

#### In vivo treatment group

After 28 and 35 days post-infection with 35 cercariae separately, mice with depilated abdomens were divided into four groups, each consisting of six mice: (a) the S-VCL-S3 recombinant collagen hydrogel injection group, (b) the praziquantel injection group, (c) the praziquantel intragastric administration group, and (d) the S-VCL-S3 recombinant collagen hydrogel containing praziquantel injection group. Praziquantel was administered at a dose of 300 mg/kg body weight via gavage or subcutaneous injection. Recombinant collagen hydrogel, praziquantel, and recombinant collagen hydrogel containing praziquantel were injected subcutaneously. After 42 days of infection, all mice in different groups were sacrificed by dislocating the cervical vertebrae. The recombinant collagen hydrogel without embedded drugs was used as the control. The portal and mesenteric veins were dissected to count the adult worms, and the worm reduction rate was calculated as follows:


$$\text{ Worm reduction rate }(\%)=\frac{\text{ average worm number of control group-average worm number of experimental group }}{\text{ average worm number of control group }}\times 100\%$$

The liver was weighed, and 10% KOH solution was added 10 times to the samples to grind the liver. Then, 50 µL homogenate solution was taken, counted the number of eggs under the microscope and calculated the average number of eggs and egg reduction rate per 5 mg of the liver were as follows:


$$\text{ Egg reduction rate }(\%)=\frac{\text{ average eggs number of control group-average eggs number of experimental group }}{\text{ average eggs number of control group }}\times 100\%$$

### Statistical analysis

The data used in this study are all expressed as mean ± standard deviation. The chi-square test (SPSS 20.0 software) was used to compare the worm and egg reduction rates of two or more independent samples. *P* < 0.05 was considered statistically significant.

## Data Availability

The data presented in this study are available in the article or supplementary material.

## SUPPLEMENTAL MATERIAL

Supplemental figures and tables spectrum.03771-23-s0001.docx Fig. S1; Tables S1 to S4.

ASM does not own the copyrights to Supplemental Material that may be linked to, or accessed through, an article. The authors have granted ASM a non-exclusive, world-wide license to publish the Supplemental Material files. Please contact the corresponding author directly for reuse.
