# Supplementary material for: A novel sustained-release agent based on disulfide-induced recombinant collagen hydrogels for the prevention and treatment of Schistosoma infections
Source: Microbiol Spectr. 2024 Dec 19;13(2):e03771-23. doi: 10.1128/spectrum.03771-23 (PMC11792459; doi:10.1128/spectrum.03771-23)
Supplement: Supplemental figures and tables — Fig. S1; Tables S1 to S4. [file spectrum.03771-23-s0001.docx]

**Table S1**. Egg and worm reduction rate of gelatin-based NCA sustained-release agent applied to the abdomen in vitro before 4-72 h *S.* *cercariae* infection (%)

| Group | Egg number | Worm number | Egg reduction rate(%) | Worm reduction rate (%) |
| --- | --- | --- | --- | --- |
| Infection rate | 56336±11231 | 20.8±2.17 | 0.00 | 0.00 |
| 72 h |  |  |  |  |
| Gelatin | 53282±13508 | 18.20±3.06 | 5.42±1.35 | 12.50±3.48 |
| Niclosamide | 24119±19619 | 8.60±2.41 | 57.19±3.71 | 58.65±5.80 |
| Anti-cercaria cream | 17303±2825 | 6.00±2.00 | 69.29±5.01 | 71.15±7.60 |
| Pa |  |  | P<0.0001 | P<0.0001 |
| Pb |  |  | P<0.0001 | P<0.0001 |
|  |  |  |  |  |
| 24h |  |  |  |  |
| Gelatin | 51289.68±3570 | 17.40±2.30 | 8.96±6.08 | 16.35±3.60 |
| Niclosamide | 20967.34±5218 | 4.80±1.15 | 62.78±3.63 | 76.92±2.56 |
| Anti-cercaria cream | 6791.00±5686 | 0.40±0.58 | 87.95±7.68 | 98.08±2.63 |
| Pa |  |  | P<0.0001 | P<0.0001 |
| Pb |  |  | P<0.0001 | P<0.0001 |
|  |  |  |  |  |
| 4 h |  |  |  |  |
| Gelatin | 40060±10585 | 15.60±3.93 | 28.89±5.42 | 25.00±3.48 |
| Niclosamide | 0.00 | 0.00 | 100.00 | 100.00 |
| Anti-cercaria cream | 0.00 | 0.00 | 100.00 | 100.00 |
| Pa |  |  | P<0.0001 | P<0.0001 |
| Pb |  |  | P>0.05 | P>0.05 |

Pa and Pb represent the pairwise comparison between the niclosamide-containing gelatin group and the gelatin group and the pairwise comparison between the niclosamide-containing gelatin group and the anti-cercaria cream group, respectively.

**Table S2.** Egg and worm reduction rate of recombinant collagen hydrogel NCA sustained-release agent applied to the abdomen in vitro before 4-72 h *S.* *cercariae* infection (%)

| Group | Egg number | Worm number | Egg reduction rate(%) | Worm reduction rate(%) |
| --- | --- | --- | --- | --- |
| Infection rate | 56350±10340 | 20.8±2.00 | 0.00 | 0.00 |
| 72 h |  |  |  |  |
| Recombinant collagen hydrogel | 55385±14849 | 19.60±9.07 | 1.69±1.35 | 5.77±5.48 |
| Recombinant collagen hydrogel containing niclosamide | 24080±19619 | 7.00±6.22 | 57.26±7.71 | 66.35±4.80 |
| Anti-cercaria cream | 17302±2825 | 6.00±2.00 | 69.29±5.01 | 71.15±7.60 |
| Anti-cercaria cream+Hydrogel-niclosamide | 11350±5486 | 3.80±0.5 | 79.85±2.85 | 81.73±2.15 |
| Pa |  |  | P<0.0001 | P<0.0001 |
| Pb |  |  | P<0.0001 | P>0.05 |
| Pc |  |  | P<0.0001 | P>0.05 |
| 24 h |  |  |  |  |
| Recombinant collagen hydrogel | 49290±18481 | 17.00±2.65 | 12.51±7.08 | 18.27±7.60 |
| Recombinant collagen hydrogel containing niclosamide | 16363±2616 | 3.80±2.63 | 70.95±4.64 | 81.73±2.40 |
| Anti-cercaria cream | 6791±5685 | 0.40±0.58 | 87.95±7.68 | 98.08±2.63 |
| Anti-cercaria cream+hydrogel-niclosamide | 5549±6866 | 0.00±0.00 | 100.00 | 100.00 |
| Pa |  |  | P<0.0001 | P<0.0001 |
| Pb |  |  | P<0.0001 | P>0.05 |
| Pc |  |  | P<0.0001 | P>0.05 |
|  |  |  |  |  |
| 4 h |  |  |  |  |
| Recombinant collagen hydrogel | 41060±34345 | 16.40±4.93 | 27.11±7.42 | 21.15±5.48 |
| Recombinant collagen hydrogel containing niclosamide | 0.00 | 0.00 | 100.00 | 100.00 |
| Anti-cercaria cream | 0.00 | 0.00 | 100.00 | 100.00 |
| Anti-cercaria cream+hydrogel-niclosamide | 0.00 | 0.00 | 100.00 | 100.00 |
| Pa |  |  | P<0.0001 | P<0.0001 |
| Pb |  |  | P<0.0001 | P<0.0001 |
| Pc |  |  | P<0.0001 | P<0.0001 |

Pa, Pb, and Pc represent the pairwise comparison between recombinant collagen hydrogel containing niclosamide and recombinant collagen hydrogel group, the pairwise comparison between recombinant collagen hydrogel containing niclosamide and anti-cercaria cream group, and the pairwise comparison between recombinant collagen hydrogel containing niclosamide and a combination of anti-cercaria cream group and hydrogel-niclosamide group, respectively.

| **Table S3.** Egg and worm reduction rate injected subcutaneously with recombinant collagen hydrogel PZQ sustained-release agent before 4 or 24 h *S. cercariae* infection (%) | | | | |
| --- | --- | --- | --- | --- |
| Group | Egg number | Worm number | Egg reduction rate(%) | Worm reduction rate(%) |
| 4 h |  |  |  |  |
| Subcutaneous injection of hydrogel | 56736±14445 | 20.4±.1.67 | 0.00 | 0.00 |
| Intragastric administration of praziquantel | 0.00 | 0.00 | 100.00 | 100.00 |
| Subcutaneous injection of praziquantel | 0.00 | 0.00 | 100.00 | 100.00 |
| Subcutaneous injection of hydrogel-praziquantel | 0.00 | 0.00 | 100.00 | 100.00 |
| Pa |  |  | P<0.0001 | P<0.0001 |
| Pb |  |  | P>0.05 | P>0.05 |
| Pc |  |  | P>0.05 | P>0.05 |
|  |  |  |  |  |
| 24 h |  |  |  |  |
| Subcutaneous injection of hydrogel | 57534±10231 | 20.4±2.00 | 0.00 | 0.00 |
| Intragastric administration of praziquantel | 30924±8771 | 3.60±1.95 | 45.11±6.65 | 82.69±8.04 |
| Subcutaneous injection of praziquantel | 28631±20158 | 2.00±0.71 | 59.18±4.35 | 90.38±3.40 |
| Subcutaneous injection of hydrogel- praziquantel | 0.00 | 0.00 | 100.00 | 100.00 |
| Pa |  |  | P<0.0001 | P<0.0001 |
| Pb |  |  | P<0.0001 | P>0.05 |
| Pc |  |  | P<0.0001 | P>0.05 |

Pa, Pb, and Pc represent the pairwise comparison between the subcutaneous injection of the hydrogel-praziquantel group and subcutaneous injection of hydrogel group, the pairwise comparison between subcutaneous injection of a hydrogel-praziquantel group and intragastric administration of praziquantel group, and the pairwise comparison between subcutaneous injection of a hydrogel-praziquantel group and subcutaneous injection of praziquantel group, respectively.

| **Table S4.** Egg and worm reduction rate injected subcutaneously with recombinant collagen hydrogel PZQ sustained-release agent after 28 or 35 days of *S. cercariae* infection (%) | | | | |
| --- | --- | --- | --- | --- |
| Group | Egg number | Worm number | Egg reduction rate(%) | Worm reduction rate(%) |
| 28 days |  |  |  |  |
| Subcutaneous injection of hydrogel | 56447±19976 | 20.00±2.55 | 0.00 | 0.00 |
| Intragastric administration of praziquantel | 33697±16402 | 4.00±1.67 | 40.18±6.47 | 80.77±5.88 |
| Subcutaneous injection of praziquantel | 23968±22921 | 3.20±1.92 | 57.46±6.60 | 84.62±9.24 |
| Subcutaneous injection of hydrogel-praziquantel | 11199±7491 | 1.00±1.00 | 80.12±8.69 | 95.19±4.81 |
| Pa |  |  | P<0.0001 | P<0.0001 |
| Pb |  |  | P<0.0001 | P>0.05 |
| Pc |  |  | P<0.0001 | P>0.05 |
|  |  |  |  |  |
| 35 days |  |  |  |  |
| Subcutaneous injection of hydrogel | 56736±14445 | 20.40±1.67 | 0.00 | 0.00 |
| Intragastric administration of praziquantel | 36198±7665 | 0.40±0.45 | 35.75±9.88 | 98.08±1.85 |
| Subcutaneous injection of praziquantel | 36759±18306 | 0.80±0.83 | 34.75±4.91 | 96.15±4.02 |
| Subcutaneous injection of hydrogel-praziquantel | 39290±9401 | 1.80±1.30 | 30.26±9.17 | 91.35±6.26 |
| Pa |  |  | P<0.0001 | P<0.0001 |
| Pb |  |  | P<0.0001 | P>0.05 |
| Pc |  |  | P<0.0001 | P>0.05 |
| Pa, Pb, and Pc represent the pairwise comparison between subcutaneous injection of hydrogel-praziquantel group and subcutaneous injection of hydrogel group, the pairwise comparison between the subcutaneous injection of the hydrogel-praziquantel group and intragastric administration of praziquantel group, and the pairwise comparison between subcutaneous injection of a hydrogel-praziquantel group and subcutaneous injection of praziquantel group, respectively. | | | | |


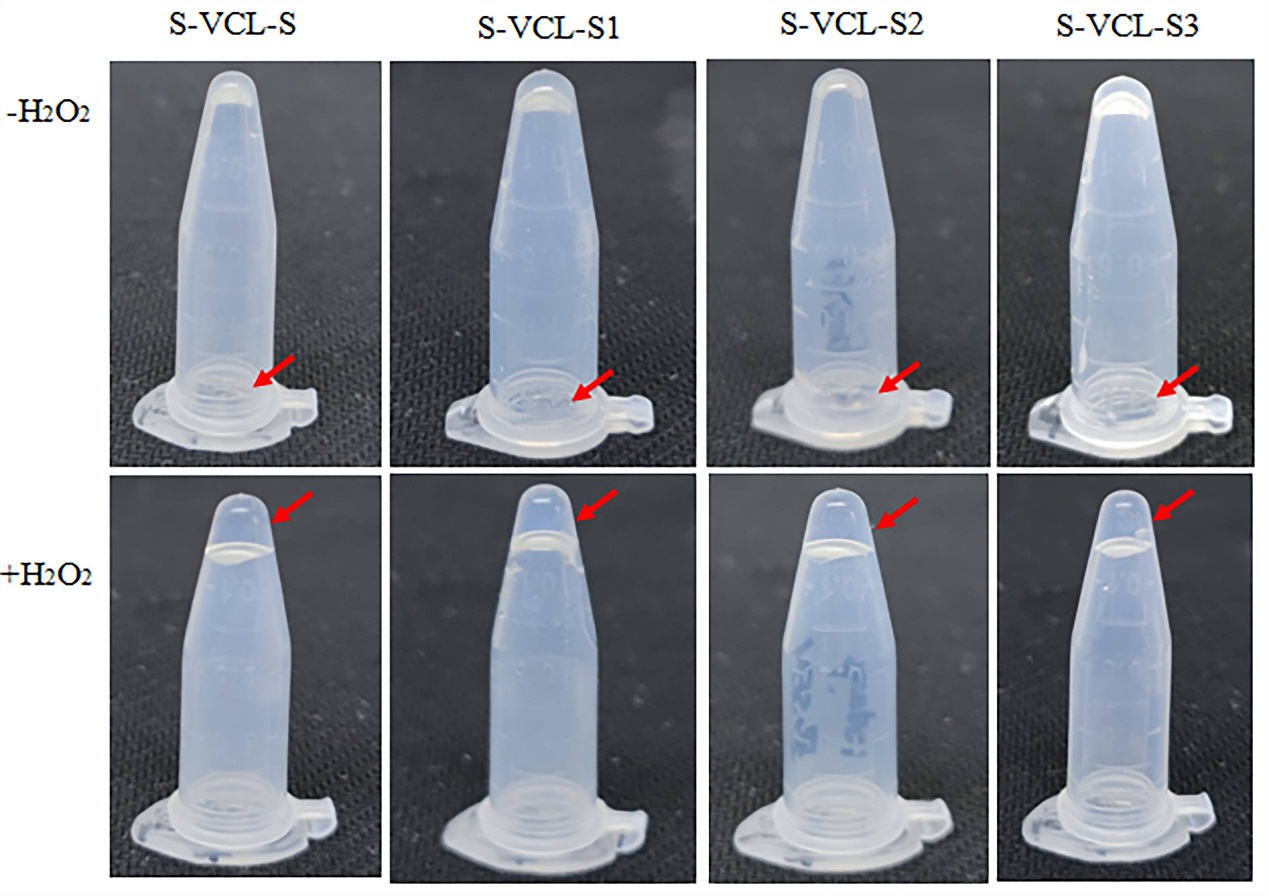


**Figure S1.** Tube inversion test of hydrogel formation under 4 wt.% of S-VCL-S, S-VCL-S1, SVCL-S2, and S-VCL-S3 with and without 0.1% H_2_O_2_.
